# Miniaturized method for the quantification of persistent organic pollutants and their metabolites in HepG2 cells: assessment of their biotransformation

**DOI:** 10.1007/s00216-023-04781-w

**Published:** 2023-06-08

**Authors:** Paloma De Oro-Carretero, Jon Sanz-Landaluze

**Affiliations:** Department of Analytical Chemistry, Faculty of Chemical Science, Complutense University of Madrid, Avenida Complutense S/N, 28040 Madrid, Spain

**Keywords:** PAHs, PBDEs, GC–MS-µECD, Metabolites, HepG2, Metabolization

## Abstract

Biotransformation can greatly influence the accumulation and, subsequently, toxicity of substances in living beings. Although traditionally these studies to quantify metabolization of a compound have been carried out with in vivo species, currently, in vitro test methods with very different cell lines are being developed for their evaluation. However, this is still a very limited field due to multiple variables of a very diverse nature. So, an increasing number of analytical chemists are working with cells or other similar biological samples of very small size. This makes it necessary to address the development of analytical methods that allow determining their concentration both inside the cells and in their exposure medium. The aim of this study is to develop a set of analytical methodologies for the quantification of polycyclic aromatic hydrocarbons, PAHs (phenanthrene, PHE), and polybrominated diphenyl ethers, PBDEs (2,2′,4,4′-tetrabromodiphenyl ether, BDE-47), and their major metabolites in cells and their exposure medium. Analytical methodologies, based on miniaturized ultrasound probe-assisted extraction, gas chromatography–mass spectrometry–microelectron capture detector (GC–MS-µECD), and liquid chromatography–fluorescence detector (LC-FL) determination techniques, have been optimized and then applied to a biotransformation study in HepG2 at 48 h of exposure. Significant concentrations of the major metabolites of PHE (1-OH, 2-OH, 3-OH, 4-OH-, and 9-OH-PHE) and BDE-47 (5-MeO-, 5-OH-, and 3-OH-BDE-47) were detected and quantified inside the cells and in the exposure medium. These results provide a new method for determination and improve information on the metabolization ratios for a better knowledge of the metabolic pathways and their toxicity.

**Graphical abstract:**

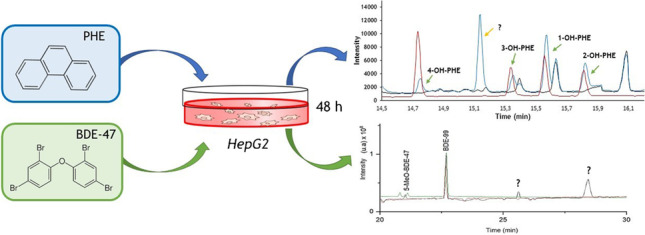

**Supplementary information:**

The online version contains supplementary material available at 10.1007/s00216-023-04781-w.

## Introduction

Persistent organic pollutants (POPs) are highly lipophilic, degradation-resistant, and bioaccumulative in the environment, organisms, and food chain compounds [1, 2]. To assess the risk to human health and the environment, the European Registration of Chemicals Regulation (REACH) classifies chemicals according to persistence, bioaccumulation, and toxicity studies during their entire life cycle, including transformation processes in the environment and metabolization. There is evidence that the bioactive metabolites [3, 4] can change the bioaccumulation and overall toxicity of the parent pollutants they come from [5–7]. In this context, analytical chemistry has become an essential part of the strategy to understand the ecotoxicity of POPs [8], through the determination of metabolites to elucidate metabolic pathways and their concentration to assess real exposure levels [7].

Most metabolization studies have been focused on analyzing tissues from in vivo experiments [1, 9, 10]. However, due to the increasing need for rapid assessment and the high cost and use of animal experimentation of official in vivo methods [3, 11], in recent years, there has been considerable interest in developing and refining alternative (in vitro) cell line tests [12–15]. In addition, other lines of research have been implemented to transfer experimental results obtained in vitro to in vivo data (in vitro to in vivo extrapolation-IVIVE) [16]. To improve in silico predictions of chemical bioaccumulation in fish, a greater understanding of all biotransformation pathways is needed to help understand all the mechanisms involved in these processes. Currently, two official guidelines have been developed to estimate the biotransformation rate using rainbow trout hepatocytes (RT-HEP) (OECD TG 319A) [17] and rainbow trout liver S9 subcellular fraction (RT S9) (OECD TG 319B) [18] to assess the bioconcentration factor (BCF). These test guidelines (TG) describe a chemical assay using a substrate depletion approach where only the determination of the starting test compound in the exposure medium is necessary, assuming that the decrease in the concentration of the parent compound is the result of biotransformation and considering that no bioaccumulation processes take place. The development of accurate and reliable analytical methods could help to overcome these approaches and determine not only the depletion on the medium of the parent compounds, but also their concentration and metabolization products in cells [19] and other similar biological samples [20, 21]. The difficulties of these determinations lie in the different physico-chemical characteristics of the compounds, the large and sometimes unknown number of metabolites, their low concentration, and the small size of the samples. Furthermore, chemicals can be differentially distributed between compartments of the in vitro assay, including the exposure medium with the globular protein bovine serum albumin [22], the plastic of the plate [23], and the headspace for some volatile substances [24]. Several publications, such as those mentioned above, determine models which estimate the real concentrations available in the cell with respect to the nominal exposure, considering the physico-chemical properties [25]. This is important to correctly assess the toxicity and kinetics metabolization of persistent organic pollutants as most of them are hydrophobic and semi-volatile. Therefore, more methods are still needed to determine the concentration of compounds and their metabolites both in the exposure medium and inside the cell.

The aim of this study is to develop analytical methodologies for the quantification of polycyclic aromatic hydrocarbons (PAHs) and polybrominated diphenyl ethers (PBDEs) as models of well-known POPs and metabolization process, with carcinogenic and endocrine-disrupting properties, respectively, and their major metabolites (OH-PAHs, OH-BDEs, and MeO-BDEs) in cells and their culture medium. Phenanthrene (PHE) was chosen because it is one of the most studied compounds in in vitro assays due to its hydrophobic and volatile properties [23–25]; and 2,2′,4,4′-tetrabromodiphenyl ether BDE-47 because of its abundance and toxicity in the environment [26]. The analytical methodologies must be extremely sensitive and selective. For selective identification and characterization of metabolic profiles, nuclear magnetic resonance (NMR) and mass spectrometry (MS) are often used [27]. To achieve sensitivity for the quantification of PBDEs and PAHs, electronic capture detection (ECD) and fluorescence detection (FL), respectively, or MS detectors in both cases, coupled with chromatographic techniques are widely used [28, 29]. Related to sample preparation, different extraction methods, such as Soxhlet, microwave, and ultrasound, have been used to extract PAHs, PBDEs, and their metabolites from environmental samples [30, 31]. Nowadays, there is a tendency to use more environmentally friendly methods, minimizing solvent volumes and sample treatment time. Taking steps in this direction and due to the small size of the samples, the analytical protocol here devised is based on miniaturized ultrasonic probe-assisted extraction. The methodology was applied to the PHE and BDE-47 biotransformation assay in human hepatoma cells (HepG2) at 48 h of exposure. HepG2 cell line was selected as a model for biotransformation studies, as metabolism of much of xenobiotics usually takes place in the liver [32].

## Material and methods

### Reagents

Individual solid standards of BDE-47, PHE, fluorene (FLU), 1-OH-PHE, 2-OH-PHE, 3-OH-PHE, and 4-OH-PHE were purchased from Sigma-Aldrich (Madrid, Spain). Individual commercial standards of BDE-28, BDE-99 in methanol, and triclosan (TCS) in acetone were purchased from Dr. Ehrenstorfer GmbH (Augsburg, Germany); individual standards of 5-MeO-BDE-47, 3-MeO-BDE-47 in methanol, 5-OH-BDE-47, 3-OH-BDE-47, and 2′-OH-BDE-28 in acetonitrile were supplied by AccuStandard Inc. (New Haven, CT, USA). The derivatization reagents N-tert-butyldimethylsilyl-N-methyltrifluoroacetamide (MTBSTFA) + 1% tert-butyl-methylchlorosilane, N,O-bis(trimethylsilyl)trifluoroacetamide (BSTFA), and N, O-Bis(trimethylsilyl)acetamide (BSA) were supplied by Sigma-Aldrich (Madrid, Spain). Analytical-grade solvents acetonitrile (ACN), chloroform, isooctane, n-hexane, dichloromethane (DCM), methyl tert-butyl ether (MTBE), dimethyl sulfoxide (DMSO), and acetone were purchased from Scharlab (Barcelona, Spain). NaCl was supplied by Sigma-Aldrich (Madrid, Spain).

Dulbecco’s Modified Eagle Medium (DMEM), penicillin (10,000 U/ml)/streptomycin (10,000 μg/mL) mixture, trypsin–EDTA (0.05%), phosphate-buffered saline (PBS), fetal bovine serum (FBS), and trypan blue 0.4% were purchased from Thermo Fisher Scientific (Spain). 3-(4,5-dimethylthiazol-2-yl)-2,5-diphenyltetrazolium bromide (MTT) was supplied by Sigma-Aldrich (Madrid, Spain).

### Instrument and apparatus

Sample preparation was carried out using a vortex mixer from Scientific Industries (NY, USA), a Vibra cell VCx130 ultrasound probe from Sonics & Materials Inc. (CT, USA) with a 2 mm diameter titanium microtip and a 130 W high-frequency generator at 20 kHz, X50S metal carbide technical nitrogen stream (Barcelona, Spain), and the Eppendorf 5415R microcentrifuge (Hamburg, Germany). Plastic and glass P100 culture plates, 96-well plates, and a CO_2_ incubator (MIDI 40) were purchased from Thermo Fisher Scientific (Spain).

Instrumental determination was performed by Agilent Gas Chromatographic Mod. 7890A Series (Agilent Technologies, Madrid, Spain) equipped with a HP 7683B autoinjector, a microelectron capture detector (μECD), a HP 5975C VL MSD mass spectrometry detector, and a polydimethylsiloxane (95%) ZB-5 capillary column (30 m × 0.25 mm I.D., 0.25-μm film thickness); and HPLC Spectra System P 4000 (Thermo Electron Corporation, San Jose, USA) equipped with a programmable fluorescence detector FL 3000 and Hypersil Green PAH columns (150 mm × 3 mm; particle size 3 μm) attached to a Phenomenex C18 pre-column.

### Cell culture

Human hepatoma cells (HepG2), provided by American Type Culture Collection, ATCC (VA, USA), were cultured in plastic P100 plates with a complete cell culture medium consisting of DMEM supplemented with 10% heat-inactivated FBS and 1% penicillin (10,000 U·mL^−1^)/streptomycin (10,000 μg·mL^−1^) mixture and maintained in a humidified incubator at 37 °C and 5% CO_2_. The medium was changed every 2 or 3 days and cells passed at 70% confluence.

### Cell viability

Cell viability was determined by MTT assay. First, 10,000 cells/well were cultured in 96-well plates for 24 h at 37 °C and 5% CO_2_. After that time, the culture medium was replaced with a culture medium containing different concentrations (0–70 mg·L^−1^) of PHE and BDE-47 for 48 h of exposure under the same incubated conditions. After that, 20 μL MTT solution (5 mg·mL^−1^ diluted in PBS) was added and incubated for the following 4 h. Viable cellular metabolic reduction of MTT produced formazan crystals, which were dissolved in 100 μL sterile DMSO and quantified with absorbance measurement at 595 nm in a Multiskan Sky High (Tecan Thermo Fisher). The cell viability was estimated by using Eq. (1).

1 $$\%\;Viability=\frac{A_{exp}-A_{blank}}{A_{control}-A_{blank}}\bullet100$$

where *A*_*control*_, *A*_*exp*_, and *A*_*blank*_ are the mean absorbance value (6 replicates) of the control group (cells without pollutant), experimental group, and blank group (only cell culture medium), respectively. Five independent assays were carried out with the same conditions. The results have been subsequently used for the design of the biotransformation experiment.

### Biotransformation experiment

Exposure of HepG2 cells (3 replicates) was performed by initially seeding approximately 2·10^6^ cells in glass P100 plates and being incubated for 24 h at 37 °C (5% CO_2_) with 10 mL of DMEM supplemented with 10% FBS. The culture medium was replaced with contaminated medium (15 mg·L^−1^ for PHE and 20 mg·L^−1^ for BDE-47) and incubated for 48 h under the same conditions. The nominal exposure concentration was estimated by the previous MTT viability study, in which, more than 70% of the cells were alive at the end of the test (lethal concentration 30, LC_30_) to ensure good metabolizing capacity. Part of the contaminated medium was stored to determine the concentration at time 0 of exposure (“*t* = 0 h”). In addition, to consider possible analyte loss, a plate of control cells without pollutant (“blank”) and a plate with contaminated medium without cells (“no cells”) were incubated under the same conditions. After 48 h of incubation, the exposure medium of each sample was stored in glass vials at − 4 °C until analysis. Since the determination of PHE is carried out by LC-FL and its metabolites by GC–MS, the cell pellet was divided in half for each analysis. For this purpose, the adherent cells were lifted in suspension by trypsinization, and the total volume of the homogenous cell suspension was divided in half. Then, the resulting cell pellet from each fraction was collected by centrifuging at 1500 rpm for 5 min and removing the medium. However, for the determination of BDE-47 and its metabolites, the complete pellet obtained is analyzed because a simultaneous extraction is performed for both analyses. The obtained pellet was dried under a gentle stream of nitrogen, weighed on a precision analytical balance (± 0.00001 g), and stored at − 4 °C until analysis. Three independent assays were carried out with the same conditions, each with 3 replicates (plates) of the same concentration.

### Analytical procedure

#### Instrumental determination

HPLC-FL and GC–MS were used for the determination of PHE and its OH-PHE metabolites (1-OH-PHE, 2-OH-PHE, 3-OH-PHE, and 4-OH-PHE) respectively. For PHE and FLU (used as internal standard, IS) separation, the mobile phase was made up of ACN and water, increasing from 70:30 ratio to 100:0 over 7 min and recovering to initial conditions at 15 min, with 0.3 mL·min^−1^ constant flow rate. Twenty microliters of the sample was injected into the column at 30 °C and fluorescence wavelengths used were 276 nm for excitation and 346 nm for emission. For the separation and determination of OH-PHE metabolites in the GC–MS, samples (1 μL) were injected in splitless mode at 280 °C. Helium was used as carrier gas at a constant pressure of 25 psi. The temperature of the column was programmed to increase from 80 °C (2 min) to 150 °C (2 min) at a rate of 30 °C·min^−1^, then to 250 °C (2 min) at 20 °C·min^−1^ and finally to 270 °C (2 min) at 10 °C·min^−1^. The temperature of the ion source and the transfer line of the mass spectrometer was set at 250 and 270 °C, respectively, and 70 eV for the electron beam.

The GC–MS-µECD system was used for the determination of BDE-47, its metabolites (BDE-28, 6-MeO-BDE-47, 5-MeO-BDE-47, 3-MeO-BDE-47, 5-OH-BDE-47, 3-OH-BDE-47, and 2′-OH-BDE-28), BDE-99 (used as IS for BDEs and MeO-BDEs), and TCS (IS for OH-BDEs). The MS was used for the identification and the µECD for their quantification. Both detectors work simultaneously thanks to the use of a flow splitter, placed at the end of the chromatographic column. Injection and MS conditions were the same as previously used for OH-PHE determination. Column temperature was programmed to increase from 130 °C (2 min) to 230 °C (2 min) at a rate of 25 °C·min^−1^ and then to 280 °C (2 min) at 2 °C·min^−1^. The μECD temperature was set at 320 °C and nitrogen was used as makeup gas (30 mL·min^−1^). The flow ratio of the splitter was 1:1 thanks to the additional helium input and the restrictors length of each detector according to the manual supplied by Agilent [33]. SCAN mode with standards for each analyte and mass spectra displayed in the libraries of the NIST databases and by bibliography [34, 35] were used to establish the *m/z* values for the SIM program of both GC–MS methods (Tables S1 and S2). A double determination was carried out for BDE-47 and its metabolites, directly injected into the GC–MS-µECD for determination of PBDEs and MeO-BDEs. Under the same chromatographic method, a derivatization step was needed for OH-PBDEs.

#### Sample preparation

To obtain the concentration of the analytes from the cell pellet and the exposure medium, an optimization of the sample preparation was carried out (see “Setting the analytical procedure”), testing various extraction solvents, derivatizing agents, and conditions for the different compounds in fortified samples of known concentration. Finally, the most optimal variables used for the experiment are shown below.

In the PHE experiments, for PHE quantification in cells, half of the pellet obtained (~ 5 mg) was extracted with 400 μL of ACN and sonicated with an ultrasound probe for 1 min at 30% of amplitude and a pulse on/off:2 s/5 s. On the other hand, 50 μL of exposure medium was extracted with 400 μL of ACN (+ 50 mg NaCl) with vortex for 30 s. Internal standard FLU was previously added to the extraction step to both cells and exposure medium. The mixture was centrifuged for 10 min at 10,000 rpm and the organic extract was directly injected into the LC-FL. The other half of the pellet was obtained (~ 5 mg) and 500 μL of the medium was used for OH-PHE quantification. In this case, the same extraction method was performed but using 500 μL of the hexane:DCM (1:1) mixture as a solvent. No IS was used for OH-PHE quantification as explained in the “Results and discussion” section, but recuperation studies were carried out to check losses. After the centrifugation step, the remaining extracts were evaporated to dryness under a gentle stream of nitrogen and reconstituted in 100 μL of hexane. Finally, 80 μL of the volume obtained was derivatized with 20 μL of MTBSTFA at 60 °C for 30 min in the oven and injected into GC–MS.

In the BDE-47 assay, extraction for BDE-47 and its metabolites was simultaneously performed. The entire pellet obtained (~ 10 mg) and 500 μL of exposure medium were extracted with 500 μL of hexane:MTBE mixture (1:1) under the same conditions (sonication and centrifugation) as the PHE assay. The IS BDE-99 and TCS were previously added. The organic phase was separated, and the extraction step was repeated. The remaining extracts were evaporated to dryness under a gentle stream of nitrogen and reconstituted in 100 μL isooctane. Finally, 80 μL of the volume obtained was derivatized with 20 μL of MTBSTFA at 70 °C for 30 min in the oven and injected into GC-μECD-MS for the OH-BDE quantification. PBDEs and MeO-BDEs were determined by direct injection of the remaining volume of isooctane extracts into the GC-µECD-MS because PBDEs are not stable after the derivatization process.

## Results and discussion

### Setting the analytical procedure

#### Phenanthrene and metabolites

For the PHE determination, HPLC-FL was used instead of GC–MS as it has higher sensitivity [29] and due to the different concentrations of PHE and its metabolites in the experiment that make it difficult to perform a simultaneous determination by GC–MS. Sensitive co-determination wavelengths for PHE and FLU, used as an internal standard (IS), were selected based on the literature [36, 37]. For OH-PHE metabolite determination, two different IS were proven (9-OH-FLU and triclosan) but 9-OH-PHE did not show good sensitivity and was not stable with the derivatizing reagent MTBSTFA and triclosan did not show the same recoveries with the extraction solvent which was better for OH-PHE. Because no IS was used in each analysis run, a parallel extraction and detection of fortified samples with known concentrations (high and low) of the study analytes was performed to check if there is a loss in the determination.

**Fig. 1:**
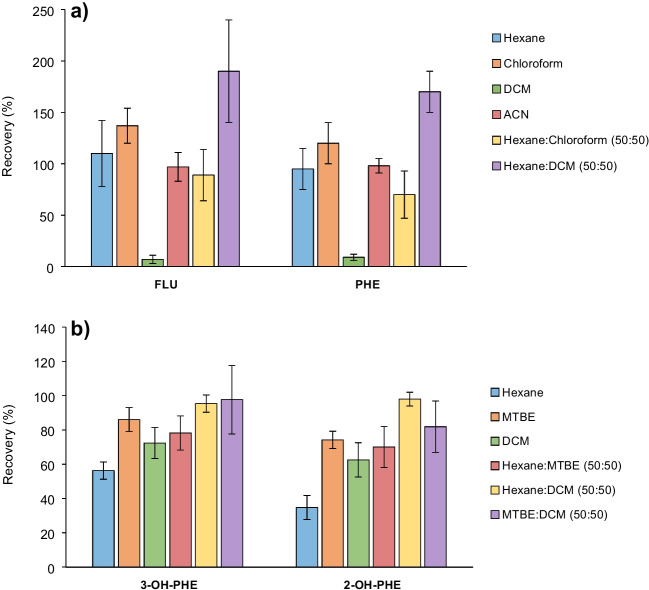
Recoveries obtained with the different extractants in cell samples of known concentration (20 µg·L^−1^) for **a** PHE and **b** OH-PAH determination. Similar behavior in the exposure media was observed

Several solvents were evaluated over fortified cells and medium samples for an optimal PHE extraction: hexane, DCM, ACN, chloroform, hexane:DCM (50:50), and hexane:chloroform (50:50). Figure 1a shows the highest extraction recoveries for both PHE and FLU when chloroform and hexane:DCM (50:50) were used as extractant; however, values well above 100% were obtained due to sample interferences being extracted. So, the best recovery results were obtained with ACN and hexane as extractants. Finally, based on recovery values and the cleaning of the extracts, ACN was chosen as an extractant agent, as they can be injected directly into HPLC. ACN is miscible with aqueous samples, so this was overcome by adding a small amount of NaCl to facilitate the separation of the aqueous and organic phases. It was proved that the volume of the medium sample was a variable in improving recovery. Higher recovery was obtained with 500 µL (Fig. S1); however, 50 µL was chosen for better interpolation on the calibration line without prior dilution of the extract.

GC–MS was used for unambiguous identification and quantification of the OH-PHEs with a prior derivatization step. The optimization process was performed by only using two metabolites (2-OH-PHE and 3-OH-PHE); however, the rest of the OH-PHEs showed the same behavior. Silylation is the most used derivatization method for OH-PAHs with derivatization reagents such as N-methyl-N-(trimethylsilyl) trifluoroacetamide (MSTFA), BSTFA, MTBSTFA, and BSA [34]. So, MTBSTFA, BSTFA, and BSA were tested at different temperatures and derivatization times looking for better derivatization conditions (Figs. S2 and S3). MTBSTFA was selected because it gave a higher analytical response and more stability, as Schummer et al. [38] also concluded. As the derivatization temperature rises, the analytical response considerably decreases and increases slightly over time. The best area/time ratio was found at 60 °C and 30 min. All injected solutions were prepared in hexane because, as shown in Fig. S4, it provided a better analytical response compared to other solvents tested. Once chromatographic conditions were optimized, several solvents were evaluated over fortified cells and medium samples: hexane, DCM, MTBE, and a mixture of them. Figure 1b shows the highest extraction recoveries for medium and cell samples when hexane:DCM (1:1) was used as the extractant solvent.

#### BDE-47 and metabolites

**Fig. 2:**
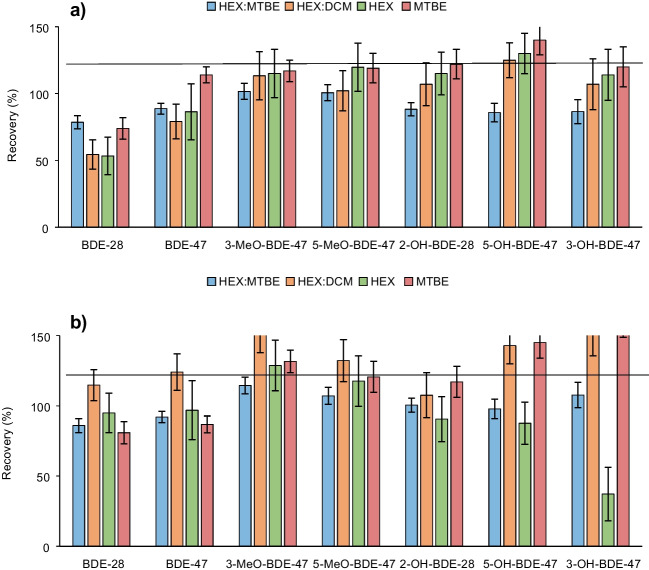
Recoveries obtained with the different extractants in **a** cells and **b** medium samples of known concentration (20 µg·L.^−1^)

GC–MS-µECD system was used for PBDE, MeO-BDE, and OH-BDE determination. MTBSTFA was chosen as a derivatization reagent because it has been shown to be better for the determination of phenolic compounds [38]. When different solvents were tested, no differences were found; however, a significant loss of PBDEs and MeO-BDEs was observed in the derivatization process (Fig. S5). So, a double determination was carried out (see “Sample preparation”), one for PBDEs and MeO-BDEs without the derivatization step and the other for OH-BDEs by derivatization resulting in two chromatograms with the same separation conditions where the compounds do not overlap (Fig. S6). For the extraction setting, several solvents were evaluated over fortified samples for an optimal simultaneous extraction of BDE-47 and their metabolites: MTBE, hexane, and a mixture of them and hexane:DCM (Fig. 2). Finally, hexane:MTBE (1:1) was chosen as the extractant for a simultaneous extraction of PBDEs, MeO-BDEs, and OH-BDEs with a consensus among the analytes of different polarity based on good recoveries and clean chromatogram and to reduce the cost and time of sample treatment.

### Method validation: QA/QC

Plastic materials were not used to avoid contaminants bonding to this material. Therefore, Hamilton syringes were always used for volume addition. All reused glass material was washed with acetone, rinsed with deionized water, and left overnight in the oven at 250 °C. The culture materials, such as glass plates, were previously sterilized with wet heat. The developed method was assessed in terms of linearity, detection and quantification limits, recoveries, and selectivity (Table S3). Selectivity was tested using several blanks for each sample type. A high degree of linearity was obtained in all analytes (*R*^2^ > 0.997). The limits of detection (LODs) and quantification (LOQ) were evaluated by integrating the result of the sum of three (LOD) or ten (LOQ) times the standard deviation of the target signal into the calibration curve. LOD values were 1.7 μg·L^−1^ and 1.0 μg·L^−1^ for PHE and BDE-47 respectively and between 1.9–2.6 μg·L^−1^ and 0.5–1.7 μg·L^−1^ for its metabolites. LOQ values were 3.1 μg·L^−1^ and 2.1 μg·L^−1^ for PHE and BDE-47 respectively and between 4.8–5.8 μg·L^−1^ and 0.9–2.0 μg·L^−1^ for its metabolites. To assess recoveries and as no certified reference material was available, sample fortification was used. Thus, higher recoveries of 83 and 87% were obtained in the determination of PHE and its metabolites for cell and medium samples, respectively. Eighty-four percent and 72% percentage recoveries were obtained in BDE-47 and its metabolites determination.

### Optimization of the cell exposure process

A major problem in in vitro cell assays is the loss of compound during the exposure process to estimate the effective concentration available to the cell and to correctly assess the toxicity and metabolization kinetics of POPs, as most of them are hydrophobic and semi-volatile [22–25]. Firstly, when concentration was determined in the exposure and *no cells* medium samples at the end of the test (48 h) using plastic Petri dishes, a loss of 89% and 84% compared to the *t* = 0 h sample was found in the cells and *no cells* sample respectively (Table 1). This loss, similar to that found in previous studies [23, 24], is mainly explained by absorption into polystyrene, the main component of in vitro culture plates [39] or evaporation processes because these compounds have a Henry law constant (*K*_H_) between 1 and 10 Pa·m^3^·mol^−1^ [11] (7.90 Pa·m^3^·mol^−1^ for PHE at 35 °C [13] and 3.78 Pa·m^3^·mol^−1^ for BDE-47 at 40 °C [40]). The remaining concentration in the medium that had not been lost by evaporation or absorbed into the cells was retained in the protein serum [22]. The difference in concentration loss between samples with and without cells indicates that is practically the same loss and that only 4 ± 1% of PHE has been taken up by the cells, which makes it difficult to appreciate differences in behavior between the experiments set out. A fraction dissolved in the medium accessible to the cells of 3.5% for anthracene (log *K*_ow_ 4.5) was obtained introducing the conditions of this work into the chemical partitioning model developed by Fischer et al. [41]. Schreiber et al. [39] compared the binding of phenanthrene on different polymers and showed that, while 94% of the initial concentration was absorbed on polystyrene, the main component of in vitro culture plates, when glass is used this absorption decreases to only 2%. Therefore, it should be considered to avoid the use of plastic in tests that requires P100 plates with a very high surface area/volume ratio of the medium when studying neutral chemicals with a log *K*_ow_ > 3, as is the case of PHE (log *K*_ow_ 4.46 [39]) and BDE-47 (log *K*_ow_ 6.81 [26]).

**Table 1:** PHE concentration in plastic and glass plate exposure test. M1, M2, and M3 are three independent experiments with cells and contaminated medium

Sample of contaminated culture medium	Conc. PHE Plastic plate exposure (mg·L^−1^)	Conc. PHE Glass plate exposure (mg·L^−1^)
*t* = 0 h	5.57	4.37
M1 – 24 h	-	2.74
M2 – 24 h	-	2.97
M3 – 24 h	-	2.89
*No cells* – 24 h	-	3.69
M1 – 48 h	0.57	2.21
M2 – 48 h	0.73	2.47
M3 – 48 h	0.62	2.37
*No cells* – 48 h	0.88	3.16

Using glass plates, a loss of 47% and 26% for cells and *no cells* samples, respectively, was found at the end of the assay (48 h) (Table 1), and the concentration absorbed by the cells increased from 4 ± 1 to 18 ± 3%. For BDE-47, a loss of only 14% because of volatilization and an absorbed concentration of 24 ± 2% resulted using a glass plate, as BDE-47 is less volatile and more hydrophobic than PHE, favoring cell uptake [42]. Birch et al. [24] calculated a cell-free fraction of 13% for PHE in HepG2 cells with 10% of FBS in the medium considering the evaporation losses using glass vials. Between 8.0 and 14.6% of the polychlorinated biphenyl (PCB) concentration with log *K*_ow_ 6.2–7.3 were recovered from HepG2 cell pellets at 48 h of exposure using plastic plates and culture medium with 10% of FBS by Zhang et al. [43]. So, considering all this, losses and assay conditions using glass plates can be considered acceptable for the metabolization experiment.

### Cell viability

**Fig. 3:**
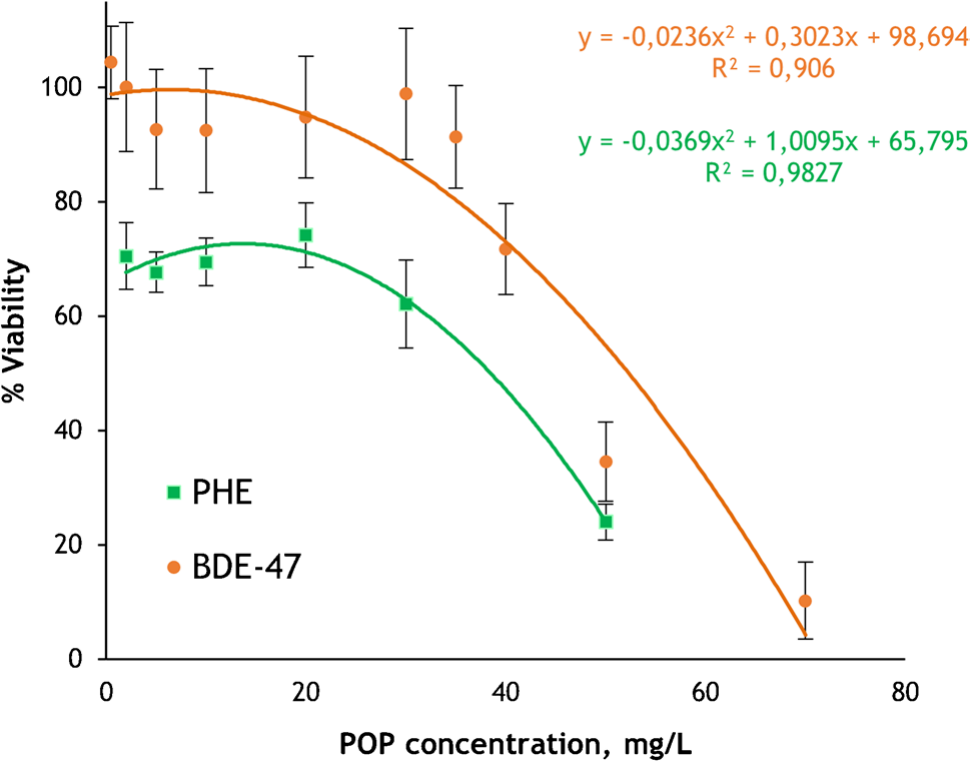
Cell viability of different PHE and BDE-47 concentration in HepG2 cells at 48 h of exposure

Cell viability of each concentration was estimated by using Eq. (1) according to the absorbance values obtained in the MTT assay. The dose–response values were fitted by Origin Pro 2021 with a polynomial equation (shown in Fig. 3) from which the LC value was estimated. The cytotoxicity of different PHE and BDE-47 concentrations in HepG2 cells at 48 h of exposure is shown in Fig. 3. LC_50_ (50% viability) and LC_30_ (70% viability) were estimated by the polynomial fitting of the mean cell viability data obtained from three independent experiments. Cell viability in HepG2 decreased by 30% at 22 mg·L^−1^ PHE and 38 mg·L^−1^ BDE-47 and up to 50% at 38 mg·L^−1^ PHE and 46 mg·L^−1^ BDE-47. Branco et al. [44] obtained a LC_50_ value of 114 µM (20.3 mg·L^−1^) in HepG2 at 48 h of PHE exposure and Liu et al. [45] and Hu et al. [46] of 100 µM (48.6 mg·L^−1^) in the case of BDE-47. The calculated LC_30_ values have been subsequently used for the design of the biotransformation experiments.

### Biotransformation in HepG2

#### Phenanthrene

**Fig. 4:**
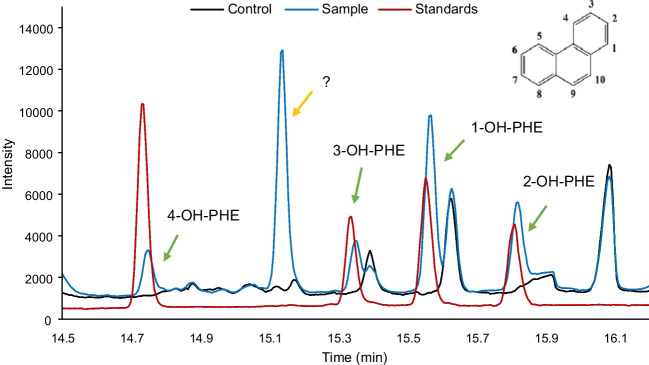
Chromatogram of medium sample obtained after 48 h of exposure to 15 mg·L.^−1^ PHE in HepG2

After 48 h of HepG2 exposure to 15 mg·L^−1^ of PHE, significant concentrations of 1-OH, 2-OH, 3-OH, 4-OH-PHE, and an unidentified compound were observed both inside and outside (exposure medium) the cell (Fig. 4 and Table 2). In control samples (*blank*, *no cells*, and *t* = 0 h), no concentrations (“n.d.”) above the limits of detection and quantification of any metabolite were detected. The ratio between metabolites and PHE was also determined (Table 2).

**Table 2:** Concentration found in culture medium and cells at 48 h. Biotransformation ratio between metabolites and PHE of three individual experiments (*n.d.* no detected)

	PHE	4-OH-PHE	3-OH-PHE	1-OH-PHE	2-OH-PHE
Concentration					
Medium	(mg·L^−1^)	(µg·L^−1^)			
Samples	5.7 ± 0.7	5 ± 1	4.0 ± 0.7	7.7 ± 0.9	5.2 ± 0.9
*No cells*	8 ± 2	n.d	n.d	n.d	n.d
*t* = 0 h	12 ± 1	n.d	n.d	n.d	n.d
Cells	(µg·g^−1^)	(ng·g^−1^)			
Samples (*n* = 3)	521 ± 144	260 ± 85	195 ± 33	72 ± 27	284 ± 92
Biotransformation ratio					
Medium	-	0.16	0.13	0.22	0.15
Cells	-	0.05	0.04	0.01	0.06

Metabolism of xenobiotics usually takes place mainly in the liver [32]. HepG2 cells have a large amount of cytochrome P450 (CYP) enzymes that enable phase I (activation) and phase II (conjugation) reactions involved in the metabolism of PAHs [47]. The first step is oxidation to form reactive epoxide intermediates, followed by hydrolysis (epoxide hydrolase) to produce hydroxylated metabolites (OH-PAHs) [48], being more soluble in water, so more easily excreted (detoxification process) [49]. These processes could explain why the metabolization ratios found in the exposure medium were higher than the ratios obtained in cells. To the best of the authors’ knowledge, no other articles in the literature evaluate either the metabolization ratio or the identification of metabolites of PHE in the HepG2 cell line are found. Metabolization studies have been performed with liver microsomes, rat cell lines, or in vivo [50]. According to these results found in the literature, major PHE metabolites transformed by the CYP 450 are 1-, 2-, 3-, 4-, and 9-OH-PHE [20], so the unidentified compound shown in Fig. 4, with an elution time of 15.1 min, is proposed to be metabolite 9-OH-PHE. The commercial standard was not available for its quantification or confirmation by retention time; however, Gaudreau et al. [48] obtains the same elution order. In addition, the *m/z* and mass spectra match this compound. Very intense 9-OH and 1-OH-PHE peaks were achieved, confirming previous results that use these metabolites as PAHs and phenanthrene inhalation biomarkers [49].

Considering the masses obtained for the parent compound and the metabolites in the medium and in the cells, a mass balance has been performed according to Eqs. (2) and (3), where a recovery of 78 ± 4% was shown at 48 h compared to 0 h. This confirms that the monohydroxy compounds formed are the major metabolites as the balance closes almost completely. It must be considered that a large part of the remaining percentage may be represented by the mass of the metabolite 9-OH-PHE, not quantified in this work, being one of the majority congeners formed. Therefore, analytical methodologies developed in this work are suitable for the quantification of metabolites in cells and their exposure media. On the other hand, although the major function of the liver is detoxification in the form of OH-PHEs, a part of them can still be reacted and hydrolyzed with the same enzymatic sequence, so that diols are formed. For this, they first activate in the form of epoxide diols, which can be coupled to endogenous compounds such as sulfuric acid, glucuronic acid, and glutathione in phase II [49]. All these intermediates, although formed in smaller proportions, also represent the unquantified percentage in the mass balance. These PAH metabolic intermediates show genotoxic and carcinogenic properties, so it would be important for future studies to develop a method of identification and quantification of each of them like this work.

2 $$m_{Total}={mPOP}_{0h}={{(mPOP}_{medium})}_{48h}+{\;{(mPOP}_{cells})}_{48h}{{+\;(mPOP}_{loss})}_{48h}+{{\sum mMetabolites}_{medium})}_{48h}+{{\sum mMetabolites}_{cells})}_{48h}$$

3 $${{(mPOP}_{loss})}_{48h}={{(mPOP}_{medium\;without\;cells})}_{48h}-{{(mPOP}_{medium\;with\;cells})}_{48h}$$

#### BDE-47

**Fig. 5:**
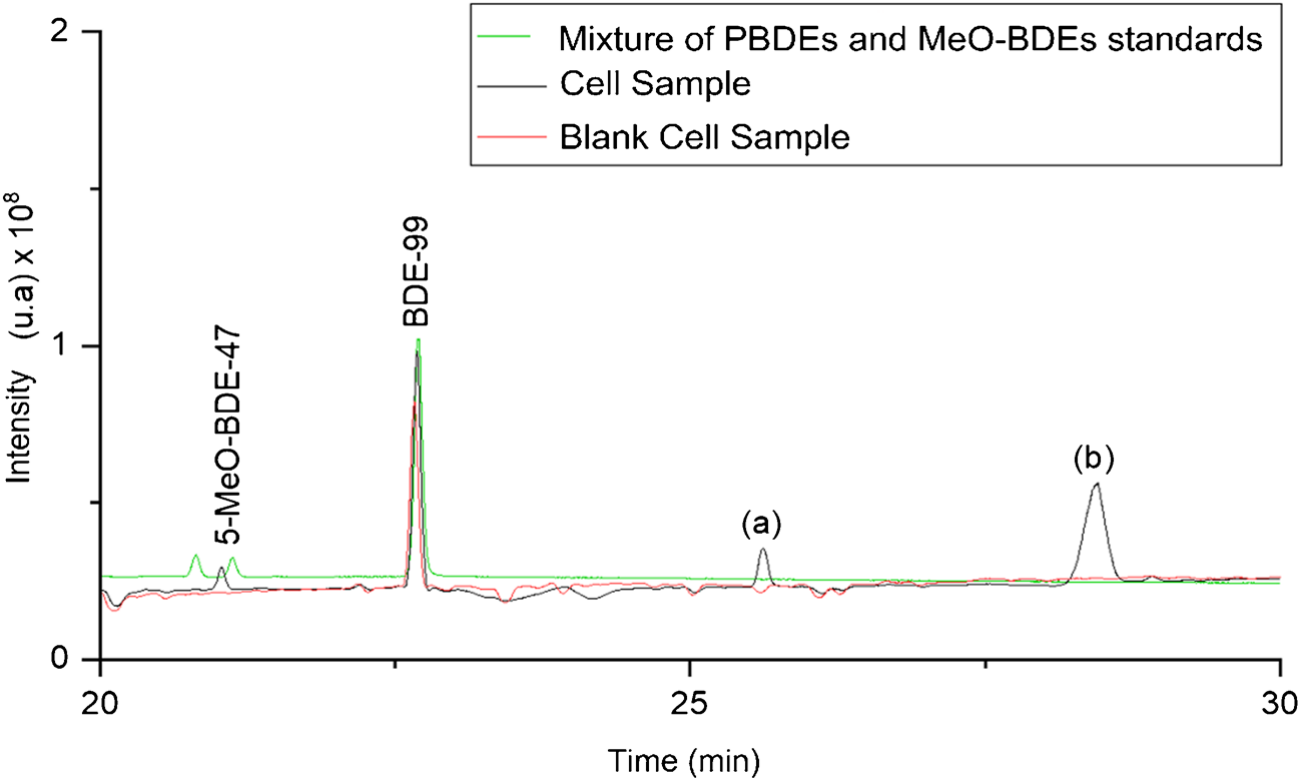
Chromatogram of cell sample obtained after 48 h of exposure to 20 mg·L.^−1^ BDE-47 in HepG2

After 48 h of exposure of HepG2 cells to 20 mg·L^−1^ BDE-47, 5-MeO-BDE-47 and two unidentified compounds at retention times of 25.6 (a) and 28.5 min (b) (Fig. 5) were observed inside the cells. 3-OH-BDE-47 and 5-OH-BDE-47 were found also in the exposure medium. The ratio between metabolites and BDE-47 concentration found in cells and medium was determined (Table 3). Biotransformation of PBDEs, as with PAHs, is carried out by liver cytochrome P450 enzymes, in which phase I enzymes introduce a polar group (OH-BDEs). In addition, several studies revealed that conversion between OH-BDEs and MeO-BDEs is possible [51]. Therefore, higher amounts of OH-BDEs are found in the culture medium when they are excreted after metabolization, while higher concentrations of MeO-BDEs were found inside the cells as their excretion is more difficult due to their lower polarity. For the same reason, the unidentified compounds are more hydrophobic metabolites and therefore they could not be removed from the environment during the experiment. A significant concentration of BDE-28 was detected in cell samples such as what we also observed in a previous zebrafish eleutheroembryo biotransformation study with these same compounds [52]. One of the major biotransformation metabolites of BDE-47 is BDE-28 by loss of bromine in the ortho position [53]. In addition, both BDE-47 and BDE-28 can be biotransformed into 2′-OH-BDE-28 [54]. However, in this case, certain amounts were also found in the control samples (*no cells* and *t* = 0 h) due to impurity of the commercial standard BDE-47 as also reported in other studies [55]. Because of that, results for BDE-28 and 2-OH-BDE-28 are not considered in this study. No quantifiable amounts of 3-MeO-BDE-47 were detected. Hu et al. [46] and Song et al. [28] showed increased expression of cytochrome P450 enzymes, involved in the metabolism of PBDEs, upon exposure to BDE-47 in HepG2 cells. To our knowledge, there are no previous studies in the literature evaluating either the metabolization ratio or the identification of metabolites in HepG2 or other cell lines.

**Table 3:** Concentration found in culture medium and cells. Biotransformation ratio between metabolites and BDE-47 of three individual experiments (*n.d.* no detected)

	BDE-47	BDE-28	3-MeO-BDE-47	5-MeO-BDE-47	2’-OH-BDE-28	5-OH-BDE-47	3-OH-BDE-47
Concentration							
Medium	(mg·L^−1^)	(µg·L^−1^)					
Samples	13 ± 2	577 ± 92	0.51 ± 0.09	1.2 ± 0.9	3.2 ± 0.2	6.2 ± 0.5	4.3 ± 0.2
*No cells*	14 ± 3	651 ± 36	n.d	n.d	n.d	n.d	n.d
*t* = 0 h	16 ± 1	662 ± 18	n.d	n.d	n.d	n.d	n.d
Cells	(µg·g^−1^)	(ng·g^−1^)					
Samples	737 ± 120	18 ± 5	n.d	0.12 ± 0.02	0.11 ± 0.09	0.27 ± 0.08	0.21 ± 0.10
Biotransformation ratio							
Medium	-	-	-	0.01	-	0.06	0.04
Cells	-	-	-	0.03	-	0.01	0.01

Considering the masses obtained for the parent compound and the metabolites in the medium and in the cells, a mass balance has been performed according to Eqs. (2) and (3), where a recovery of 87 ± 5% was shown at 48 h compared to 0 h. This confirms that the metabolites studied were the predominant biotransformed compounds. Other studies have confirmed the formation of all the metabolites studied in this work by the biotransformation of BDE-47 in bacteria [56], microalgae [57], or more developed organisms [53]. Erratico et al. [21] studied the biotransformation of BDE-47 to 6-OH-BDE-47, 5-OH-BDE-47, 3-OH-BDE-47, 2,4-DBP, and 2’-OH-BDE-28 metabolites by human liver microsomes through P450 2B6 enzymes. In addition, as with PAHs, diols can be formed [58] and can be conjugated in phase II with endogenous compounds [59]. Therefore, it would be interesting to identify and quantify other metabolites represented in the small remaining percentage of the mass balance. These could include the unquantified compounds BDE-28 and 2′OH-BDE-28, the unidentified compounds appearing in the chromatogram, and other unstudied compounds such as 6-OH-BDE-47, 6-MeO-BDE, diols, or metabolites conjugated with endogenous compounds. However, the proposed method has been shown to be suitable for the quantification of metabolites in cells and in exposure media.

## Conclusions

Two miniaturized analytical methods have been developed to determine PHE and its metabolites (OH-PHEs) and BDE-47 and its metabolites (MeO-BDEs and OH-BDEs) simultaneously. Both methods have been optimized considering different aspects: the small amount of sample, the large difference in physico-chemical properties of the analytes and their metabolites, their low concentration, and the minimum consumption of organic solvent to produce less harmful residues. The analytical techniques used give unambiguous identification and highly sensitive quantification, suitable for trace and metabolite determination due to the advantages of each detector. Therefore, the methods developed could be applied to extremely small biological samples, showing low detection limits and high reproducibility. In addition, this method has been shown to be suitable for application in metabolization studies. HepG2 cells can biotransform POPs metabolized by CYP 450 enzymes where the major metabolites of PHE and BDE-47 have been detected at 48 h of exposure inside the cells and in their exposure medium. These results indicate that compounds can be removed back to the culture medium or follow other metabolic pathways. Therefore, it would be interesting to evaluate the overall metabolization rate of the compound to know its real toxicity. Although in vitro metabolomics has already been successfully applied in a few studies using primary human hepatocytes or HepG2 [19], it would be interesting to extend this study and this method to other types of pollutants and cells to explore whether they show the same behavior.

These results may provide information on the effective concentration available to the cell relative to the nominal one and metabolization ratios for developing new methods. Also, this shown knowledge may contribute and be useful to the authors who are currently working with cells, as well as for the extrapolation data obtained in in vivo and in vitro models [60–62] to reduce animal suffering during trials. These aspects will be examined and discussed in future ecotoxicology studies through the application of the analytical method developed in this work.

## Supplementary Information

Below is the link to the electronic supplementary material.

Supplementary file1 (DOCX 290 kb)

## References

[1] Acosta-Tlapalamatl M, Romo-Gómez C, Anaya-Hernández A, Juárez-Santacruz L, Gaytán-Oyarzún JC, Acebedo-Sandoval OA, García-Nieto E (2022). Metabolomics: a new approach in the evaluation of effects in human beings and wildlife associated with environmental exposition to POPs. Toxics.

[2] Chen H, Wang C, Li HH, Ma R, Yu Z, Li L, Xiang M, Chen X, Hua X, Yu Y (2019). A review of toxicity induced by persistent organic pollutants (POPs) and endocrine-disrupting chemicals (EDCs) in the nematode Caenorhabditis elegans. J Environ Manag.

[3] Almazroo OA, Miah MK, Venkataramanan R (2021). Drug metabolism in the liver. Clin Liver Dis.

[4] Cervantes FJ, Martínez CM, Gonzalez-Estrella J, Márquez A, Arriaga S (2013). Kinetics during the redox biotransformation of pollutants mediated by immobilized and soluble humic acids. Appl Microbiol Biotechnol.

[5] Molina-Fernández N, Rainieri S, Muñoz-Olivas R, de Oro-Carretero P, Sanz-Landaluze J (2021). Development of a method for assessing the accumulation and metabolization of antidepressant drugs in zebrafish (Danio rerio) eleutheroembryos. Anal Bioanal Chem.

[6] McCormick JM, van Es T, Cooper KR, White LA, Häggblom MM (2011). Microbially mediated O-methylation of bisphenol a result in metabolites with increased toxicity to the developing zebrafish (Danio rerio) embryo. Environ Sci Technol.

[7] Fu Q, Fedrizzi D, Kosfeld V, Schlechtriem C, Ganz V, Derrer S, Rentsch D, Hollende J (2020). Biotransformation changes bioaccumulation and toxicity of diclofenac in aquatic organisms. Environ Sci Technol.

[8] Wei S, Wei Y, Gong Y, Chen Y, Cui J, Li L, Yan H, Yu Y, Lin X, Li G, Yi L (2022). Metabolomics as a valid analytical technique in environmental exposure research: application and progress. Metabolomics.

[9] Kim HM, Kang JS (2021). Metabolomic studies for the evaluation of toxicity induced by environmental toxicants on model organisms. Metabolites.

[10] Eriksson ANM, Rigaud C, Rokka A, Skaugen M, Lihavainen JH, Vehniäinen ER (2022). Changes in cardiac proteome and metabolome following exposure to the PAHs retene and fluoranthene and their mixture in developing rainbow trout alevins. Sci Total Environ.

[11] OECD. Guidance document on aquatic toxicity testing of difficult substances and mixtures. In: Organisation for Economic Co-operation and Development*.* Series on testing and assessment. Paris: OECD Publishing; 2019. 10.1787/0ed2f88e-en.

[12] Silva RJ, Tamburic SA (2022). A State-of-the-art review on the alternatives to animal testing for the safety assessment of cosmetics. Cosmetics.

[13] Kramer NI, Krismartina M, Rico-Rico A, Blaauboer BJ, Hermens JLM (2012). Quantifying processes determining the free concentration of phenanthrene in basal cytotoxicity assays. Chem Res Toxicol.

[14] U.S. National Research Council. Toxicity Testing in the 21st century: a vision and a strategy. In: Committee on Toxicity Testing and Assessment of Environmental Agents. Washington, DC: The National Academies Press; 2007. 10.17226/11970.

[15] Nichols JW, Huggett DB, Arnot JA, Fitzsimmons PN, Cowan-Ellsberry CE (2013). Toward improved models for predicting bioconcentration of well-metabolized compounds by rainbow trout using measured rates of in vitro intrinsic clearance. Environ Toxicol Chem.

[16] Bell SM, Chang X, Wambaugh JF, Allen DG, Bartels M, Brouwer KLR, Casey WM, Choksi N, Ferguson SS, Fraczkiewicz G, Jarabek AM, Ke A, Lumen A, Lynn SG, Paini A, Price PS, Ring C, Simon TW, Sipes NS, Sprankle CS, Strickland J, Troutman J, Wetmore BA, Kleinstreuer NC (2018). In vitro to in vivo extrapolation for high throughput prioritization and decision making. Toxicol In Vitro.

[17] OECD 319A. In: Determination of in vitro intrinsic clearance using cryopreserved rainbow trout hepatocytes (RT HEP). OECD Guideline for testing of chemicals. Paris: OECD Publishing; 2018. 10.1787/9789264303218-en.

[18] OECD 319B. In: Determination of in vitro intrinsic clearance using rainbow trout liver S9 sub cellular fraction (RT S9). OECD guideline for testing of chemicals. Paris: OECD Publishing; 2018. 10.1787/9789264303232-en.

[19] Ruiz-Aracama A, Peijnenburg A, Kleinjans J, Jennen D, van Delft J, Hellfrisch C, Lommen A (2011). An untargeted multi-technique metabolomics approach to studying intracellular metabolites of HepG2 cells exposed to 2,3,7,8-tetrachlorodibenzo-p-dioxin. BMC Genomics.

[20] Wang D, Schramm V, Pool J, Pardali E, Brandenburg A, Rietjens IMCM, Boogaard PJ (2022). The effect of alkyl substitution on the oxidative metabolism and mutagenicity of phenanthrene. Arch Toxicol.

[21] Erratico CA, Szeitz A, Bandiera SM (2013). Biotransformation of 2,2′,4,4′-tetrabromodiphenyl ether (BDE-47) by human liver microsomes: identification of cytochrome P450 2B6 as the major enzyme involved. Chem Res Toxicol.

[22] Kwon JH, Lee HJ, Escher BI (2020). Bioavailability of hydrophobic organic chemicals on an in vitro metabolic transformation using rat liver S9 fraction. Toxicol In Vitro.

[23] Fischer FC, Cirpka OA, Goss KU, Henneberger L, Escher BI (2018). Application of experimental polystyrene partition constants and diffusion coefficients to predict the sorption of neutral organic chemicals to multiwell plates in in vivo and in vitro bioassays. Environ Sci Technol.

[24] Birch H, Kramer NI, Mayer P (2019). Time-resolved freely dissolved concentrations of semivolatile and hydrophobic test chemicals in in vitro assays - measuring high losses and crossover by headspace solid-phase microextraction. Chem Res Toxicol.

[25] Proença S, Escher BI, Fischer FC, Fisher C, Grégoire S, Hewitt NJ, Nicol B, Paini A, Kramer NI (2021). Effective exposure of chemicals in in vitro cell systems: a review of chemical distribution models. Toxicol In Vitro.

[26] Pereira LC, de Souza AO, Meireles G, Franco-Bernardes MF, Tasso MJ, Bruno V, Dorta DJ, de Oliveira DP (2016). Comparative study of genotoxicity induced by six different PBDEs. Basic Clin Pharmacol Toxicol.

[27] Kumar B, Prakash A, Ruhela RK, Medhi B (2014). Potential of metabolomics in preclinical and clinical drug development. Pharmacol Rep.

[28] Song S, Yang C, Shao M, Chao J, Zheng N, Wang W, He Y, Li P (2020). Simultaneous determination of polybrominated diphenyl ethers and hydroxylated analogues in human serum using high-performance liquid chromatography-inductively coupled plasma mass spectrometry. J Chromatogr B Analyt Technol Biomed Life Sci.

[29] Adeniji AO, Okoh OO, Okoh AI (2018). Analytical methods for polycyclic aromatic hydrocarbons and their global trend of distribution in water and sediment: a review in Recent Insights in Petroleum Science And Engineering. INTECH.

[30] Wang X, Lin L, Luan T, Yang L, Tam NFY (2012). Determination of hydroxylated metabolites of polycyclic aromatic hydrocarbons in sediment samples by combining subcritical water extraction and dispersive liquid-liquid microextraction with derivatization. Anal Chim Acta.

[31] Cruz R, Cunha SC, Marques A, Casal S (2017). Polybrominated diphenyl ethers and metabolites – an analytical review on seafood occurrence. TrAC.

[32] Ewa B, Danuta MŠ (2017). Polycyclic aromatic hydrocarbons and PAH-related DNA adducts. J Appl Genet.

[33] Agilent Technologies. Two-way splitter kit with makeup gas installation and operation guide. Agilent G3180B, 1st edn. Wilmington, DE: Agilent Technologies, Inc; 2011. Available in: https://www.agilent.com/cs/library/usermanuals/Public/G3181-90120_045611.pdf.

[34] Motorykin O, Schrlau J, Jia Y, Harper B, Harris S, Harding A, Stone D, Kile M, Sudakin D, Staci L, Simonich M (2015). Determination of parent and hydroxy PAHs in personal PM2.5 and urine samples collected during Native American fish smoking activities. Sci Total Environ.

[35] Butryn DM, Gross MS, Chi LH, Schecter A, Olson JR, Aga DS (2015). One-shot’ analysis of polybrominated diphenyl ethers and their hydroxylated and methoxylated analogs in human breast milk and serum using gas chromatography-tandem mass spectrometry. Anal Chim Acta.

[36] Sanz-Landaluze J, Bocanegra-Salazar M, Ortiz-Pérez D, Cámara C (2010). Miniaturisated method for the analysis of polycyclic aromatic hydrocarbons in leaf samples. J Chrom A.

[37] Joseph M. HPLC detector options for the determination of polynuclear aromatic hydrocarbons varian application note number 7. 2012. www.agilent.com/chem. Accessed 6 May 2022.

[38] Schummer C, Delhomme O, Appenzeller BMR, Wennig R, Millet M (2009). Comparison of MTBSTFA and BSTFA in derivatization reactions of polar compounds prior to GC/MS analysis. Talanta.

[39] Schreiber R, Altenburger R, Paschke A, Küster E (2008). How to deal with lipophilic and volatile organic substances in microtiter plate assays. Environ Toxicol Chem.

[40] Cetin B (2005). Odabasi Measurement of Henry’s law constants of seven polybrominated diphenyl ether (PBDE) congeners as a function of temperature. Atmos Environ.

[41] Fischer FC, Henneberger L, König M, Bittermann K, Linden L, Goss KU, Escher BI (2017). Modeling exposure in the Tox21 in vitro bioassays. Chem Res Toxicol.

[42] Quinn CL, van der Heijden SA, Wania F, Jonker MTO (2014). Partitioning of polychlorinated biphenyls into human cells and adipose tissues: evaluation of octanol, triolein, and liposomes as surrogates. Environ Sci Technol.

[43] Zhang CY, Flor S, Ludewig G, Lehmler HJ (2020). Atropselective partitioning of polychlorinated biphenyls in a HepG2 cell culture system: experimental and modeling results. Environ Sci Technol.

[44] Branco V, Matos B, Mourato C, Diniz M, Carvalho C, Martins M (2021). Synthesis of glutathione as a central aspect of PAH toxicity in liver cells: a comparison between phenanthrene, Benzo[b]Fluoranthene and their mixtures. Ecotoxicol Environ Saf.

[45] Liu X, Wang J, Lu C, Zhu C, Qian B, Li Z, Liu C, Shao J, Yan J (2015). The role of lysosomes in BDE 47-mediated activation of mitochondrial apoptotic pathway in HepG2 cells. Chemosphere.

[46] Hu X, Zhang J, Jiang Y, Lei Y, Lu L, Zhou J, Huang H, Fang D, Tao G (2014). Effect on metabolic enzymes and thyroid receptors induced by BDE-47 by activation the pregnane X receptor in HepG2, a human hepatoma cell line. Toxicol In Vitro.

[47] Kang Y, Cheung KC, Wong MH (2010). Polycyclic aromatic hydrocarbons (PAHs) in different indoor dusts and their potential cytotoxicity based on two human cell lines. Environ Int.

[48] Gaudreau É, Bérubé R, Bienvenu JF, Fleury N (2016). Stability issues in the determination of 19 urinary (free and conjugated) monohydroxy polycyclic aromatic hydrocarbons. Anal Bioanal Chem.

[49] Gao P, da Silva E, Hou L, Denslow ND, Xiang P, Ma LQ (2018). Human exposure to polycyclic aromatic hydrocarbons: metabolomics perspective. Environ Int.

[50] Schober W, Pusch G, Oeder S, Reindl H, Behrendt H, Buters JTM (2010). Metabolic activation of phenanthrene by human and mouse cytochromes P450 and pharmacokinetics in CYP1A2 knockout mice. Chem Biol Interact.

[51] Zheng X, Zhu Y, Liu C, Liu H, Giesy JP, Hecker M, Lam MHW, Yu H (2012). Accumulation and biotransformation of BDE-47 by zebrafish larvae and teratogenicity and expression of genes along the hypothalamus-pituitary-thyroid axis. Environ Sci Technol.

[52] De Oro-Carretero P, Sanz-Landaluze J (2023). Bioaccumulation and biotransformation of BDE-47 using zebrafish eleutheroembryos (Danio rerio). Environ Toxicol Chem.

[53] Sun J, Liu J, Yu M, Wang C, Sun Y, Zhang A, Wang T, Lei Z, Jiang G (2013). In vivo metabolism of 2,2′,4,4′-tetrabromodiphenyl ether (BDE-47) in young whole pumpkin plant. Environ Sci Technol.

[54] Yamazoe A, Yagi O, Oyaizu H (2004). Biotransformation of fluorene, diphenyl ether, dibenzo-p-dioxin and carbazole by Janibacter sp. Biotech Lett.

[55] Wiseman SB, Wan Y, Chang H, Zhang X, Hecker M, Jones PD, Giesy JP (2011). Polybrominated diphenyl ethers and their hydroxylated/methoxylated analogs: environmental sources, metabolic relationships, and relative toxicities. Mar Pollut Bullet.

[56] Tang J, Hu B, Zheng H, Qian X, Zhang Y, Zhu J, Xu G, Chen D, Jin X, Li W, Xu L (2021). 2,2′,4,4′-Tetrabromodiphenyl ether (BDE-47) activates aryl hydrocarbon receptor (AhR) mediated ROS and NLRP3 inflammasome/p38 MAPK pathway inducing necrosis in cochlear hair cells. Ecotoxicol Environ Saf.

[57] Deng D, Tam NFY (2016). Adsorption-uptake-metabolism kinetic model on the removal of BDE-47 by a Chlorella isolate. Environ Poll.

[58] Zhang M, Zhao F, Zhang J, Shi J, Tao H, Ge H, Guo W, Liu D, Cai Z (2020). Toxicity and accumulation of 6-OH-BDE-47 and newly synthesized 6,6′-diOH-BDE-47 in early life-stages of zebrafish (Danio rerio). Sci Total Environ.

[59] De Souza AC, Sardela VF, De Sousa VP, Pereira HMG (2018). Zebrafish (Danio rerio): a valuable tool for predicting the metabolism of xenobiotics in humans?. CBPC.

[60] Laue H, Hostettler L, Badertscher RP, Badertscher RP, Jenner KJ, Sanders G, Arnot JA, Natsch A (2020). Examining uncertainty in in vitro-in vivo extrapolation applied in fish bioconcentration models. Environ Sci Technol.

[61] Yamazaki S, Evers R, de Zwart L (2022). Physiologically-based pharmacokinetic modelling to evaluate in vitro-to-in vivo extrapolation for intestinal P-glycoprotein inhibition. CPT Pharmacometrics Syst Pharmacol.

[62] Grimard C, Mangold-Döring A, Schmitz M, Alharbi H, Jones PD, Giesy JP, Hecker M, Brinkmann M (2020). In vitro-in vivo and cross-life stage extrapolation of uptake and biotransformation of benzo[a]pyrene in the fathead minnow (Pimephales promelas). Aquat Toxicol.

