# Supplementary material for: Miniaturized method for the quantification of persistent organic pollutants and their metabolites in HepG2 cells: assessment of their biotransformation
Source: Anal Bioanal Chem. 2023 Jun 8;415(19):4813–25. doi: 10.1007/s00216-023-04781-w (PMC10352165; doi:10.1007/s00216-023-04781-w)

**Miniaturized method for the quantification of Persistent Organic Pollutants and their metabolites in HepG2 cells: assessment of their biotransformation**

**Paloma De Oro-Carretero* and Jon Sanz-Landaluze**

Department of Analytical Chemistry, Faculty of Chemical Science, Complutense University of Madrid, Avenida Complutense s/n, 28040 Madrid, Spain

***Contact emails:*** pdeoro@ucm.es* (P. De Oro-Carretero), jsanzlan@ucm.es (J. Sanz-Landaluze)

***Phone:*** +034-91 394 4322; Fax: +034-91 394 4329

*****Corresponding author

**Supporting information**

**Table S1**. Mass spectrometer conditions in SIM mode for OH-PHEs

| **Analyte** | **Retention time (min)** | **m/z** | **Initial scanning time (min)** |
| --- | --- | --- | --- |
| 4-OH-PHE | 14.73 | 308, 251, 235 | 8 (solvent delay) |
| 3-OH-PHE | 15.34 |  | 18 |
| 1-OH-PHE | 15.55 |  |  |
| 2-OH-PHE | 15.80 |  |  |

**Table S2.** Mass spectrometer conditions in SIM mode for BDE-47 and their metabolites

| **Analyte** | **Retention time (min)** | **m/z** | **Initial scanning time (min)** |
| --- | --- | --- | --- |
| BDE-28 | 12.01 | 248, 246, 406, 408 | 8 (solvent delay) |
| TMDS-TCS | 13.36 | 290, 288, 218 | 12.5 |
| BDE-47 | 16.55 | 326, 486, 484, 488 | 15.50 |
| 2’-OH-TMDS-BDE-28 | 19.59 | 423, 421, 425, 81 | 19.0* |
| 3-MeO-BDE-47 | 20.83 | 356, 516, 341, 514 |  |
| 5-MeO-BDE-47 | 21.15 | 516, 356, 358, 326 |  |
| BDE-99 | 22.73 | 404, 406, 564, 566 | 22.00 |
| 5-OH-TMDS-BDE-47 | 27.20 | 502, 504, 500, 81 | 25.50 |
| 3-OH-TMDS-BDE-47 | 31.94 | 502, 474, 266, 419 | 29.50 |

* A joint window is established as they elude close retention times, so that the majority m/z of each is monitored (marked in the table)

**Table S3.** LODs, LOQs RSD, R^2^ and method recoveries.

| **Analyte** | **R^2^** | **LOD (µg·L^-1^)** | **LOQ (µg·L^-1^)** | **Recovery of cell samples (%)** | **Recovery of medium samples (%)** |
| --- | --- | --- | --- | --- | --- |
| PHE | 0.9990 | 1.71 | 3.11 | 96 ± 7 | 105 ± 11 |
| 4-OH-PHE | 0.9980 | 2.65 | 5.89 | 85 ± 9 | 87 ± 9 |
| 3-OH-PHE | 0.9984 | 2.30 | 5.30 | 86 ± 9 | 91 ± 8 |
| 1-OH-PHE | 0.9989 | 1.98 | 4.87 | 90 ± 5 | 91 ± 7 |
| 2-OH-PHE | 0.9985 | 2.52 | 5.36 | 83 ± 10 | 87 ± 10 |
| BDE-28 | 0.9997 | 0.60 | 1.25 | 91 ± 7 | 85 ± 7 |
| BDE-47 | 0.9990 | 1.01 | 2.06 | 97 ± 6 | 98 ± 5 |
| 3-MeO-BDE-47 | 0.9984 | 0.70 | 1.02 | 112 ± 3 | 92 ± 6 |
| 5-MeO-BDE-47 | 0.9991 | 0.39 | 0.68 | 105 ± 9 | 87 ± 5 |
| 2'-OH-BDE-28 | 0.9992 | 1.52 | 2.03 | 87 ± 11 | 89 ± 6 |
| 5-OH-BDE-47 | 0.9984 | 0.55 | 0.96 | 95 ± 10 | 75 ± 6 |
| 3-OH-BDE-47 | 0.9972 | 1.70 | 2.13 | 84 ± 6 | 72 ± 7 |

**Fig. S1.** Recoveries obtained with the different sample volume of medium (20 µg·L^-1^).

**Fig. S2.** Derivatized metabolites areas (20 µg·L^-1^) with different reagents for 30 min at 60 °C.

**Fig. S3.** Areas of the metabolites (20 µg·L^-1^) with different conditions of derivatization with MTBSTFA reagent.

**Fig. S4.** Areas of the derivatized metabolites (MTBSTFA - 20 µg·L^-1^) with different solvents.

**Fig. S5.** Analytical signal obtained by derivatising a mixture of OH-BDEs and a BDE in different solvents (10 µg·L^-1^).

**Fig. S6.** Chromatogram of a mixture of the analytes (10 µg·L^-1^) obtained with µECD detector (is like MS detector).


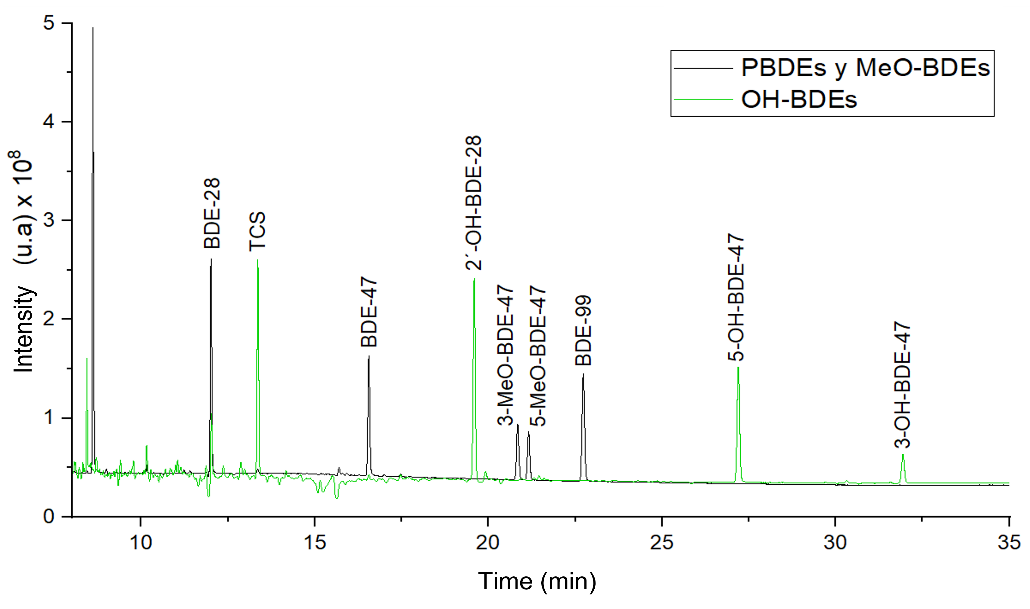

Supplement: Supplementary file 1 — Supplementary file1 (DOCX 290 kb) [file 216_2023_4781_MOESM1_ESM.docx]
